# Circulating miR-323-3p is a biomarker for cardiomyopathy and an indicator of phenotypic variability in Friedreich’s ataxia patients

**DOI:** 10.1038/s41598-017-04996-9

**Published:** 2017-07-12

**Authors:** M. Seco-Cervera, D. González-Rodríguez, J. S. Ibáñez-Cabellos, L. Peiró-Chova, P. González-Cabo, E. García-López, J. J. Vílchez, I. Sanz-Gallego, F. V. Pallardó, J. L. García-Giménez

**Affiliations:** 1 Centro de Investigación Biomédica en Red de Enfermedades Raras (CIBERER), Valencia, Spain; 2 Instituto de Investigación Sanitaria INCLIVA, Mixt Unit for rare diseases INCLIVA-CIPF, Avenida de Menéndez y Pelayo, 4, 46010 Valencia, Spain; 3 Department of Physiology, Faculty of Medicine and Dentistry, University of Valencia, Av/Blasco Ibáñez, 15, 46010 Valencia, Spain; 4 Instituto de Investigación Sanitaria INCLIVA, INCLIVA Biobank, Avenida de Menéndez y Pelayo, 4, 46010 Valencia, Spain; 5 Instituto de Investigación Sanitaria IISLAFE, Av/Fernando Abril Martorell, 106, Torre A 7, 46026 Valencia, Spain; 6 Department of Neurology Hospital Nuestra Señora de Sonsoles, Ávila, Spain

## Abstract

MicroRNAs (miRNAs) are noncoding RNAs that contribute to gene expression modulation by regulating important cellular pathways. In this study, we used small RNA sequencing to identify a series of circulating miRNAs in blood samples taken from Friedreich’s ataxia patients. We were thus able to develop a miRNA biomarker signature to differentiate Friedreich’s ataxia (FRDA) patients from healthy people. Most research on FDRA has focused on understanding the role of frataxin in the mitochondria, and a whole molecular view of pathological pathways underlying FRDA therefore remains to be elucidated. We found seven differentially expressed miRNAs, and we propose that these miRNAs represent key mechanisms in the modulation of several signalling pathways that regulate the physiopathology of FRDA. If this is the case, miRNAs can be used to characterize phenotypic variation in FRDA and stratify patients’ risk of cardiomyopathy. In this study, we identify miR-323-3p as a candidate marker for phenotypic differentiation in FRDA patients suffering from cardiomyopathy. We propose the use of dynamic miRNAs as biomarkers for phenotypic characterization and prognosis of FRDA.

## Introduction

Friedreich’s ataxia (FRDA), an autosomal recessive neurodegenerative mitochondrial disease, is the most prevalent hereditary ataxia in people of European descent, affecting around 2–5 people in every 100,000 (Orphanet reports). This rare, childhood-onset disease is characterized by a progressive loss of sensory neurons in the dorsal root ganglia (DRG) and posterior columns. This loss of neurons is followed by degeneration of corticospinal and spinocerebellar tracts of the spinal cord, culminating in gait and limb ataxia, loss of tendon reflexes and dysarthria^1, 2^. The cerebellar dentate nucleus is also affected^3^. Other non-neurological features of FRDA are scoliosis, diabetes and cardiac symptoms^4–6^. Hypertrophic cardiomyopathy, which is found in two thirds of FRDA patients at the time of diagnosis, is the primary cause of death in these patients^7, 8^.

FRDA is most often caused by a homozygous GAA repeat expansion mutation (typically between 600 and 1200 repeats) in the first intron of the frataxin gene (FXN), which is found on chromosome 9q21.11 and encodes the protein frataxin^2, 9^. The elevated number of GAA repeats and resulting blockage effects on the RNAPII transcription machinery (“sticky DNA”, hairpin structures, parallel duplex structures, R-loops, etc.; reviewed in ref. 10) have been proposed as causes of decreased expression of the mitochondrial protein frataxin^11^. The frataxin protein is nuclearly encoded, cytoplasmatically expressed and finally introduced into the mitochondria through an N-terminal import signal^12, 13^.

Previous studies have reported the involvement of the FXN protein in the mitochondrial biogenesis of iron-sulphur clusters (ISC)^14–16^. Friedreich’s ataxia is associated with mitochondrial respiratory chain dysfunction, mitochondrial iron accumulation, decreased mitochondrial DNA levels, oxidative stress, reduced generation of ATP and muscular weakness. Although most research has focused on understanding the role of frataxin in the mitochondria, a whole molecular view of pathways involved in FRDA remains to be elucidated. We have previously demonstrated the relationship between antioxidant cellular response, energy metabolism and mitochondrial signalling in fibroblasts from FRDA patients, highlighting the possible role of different metabolic sensors (AMPK, p38, PGC-1α and mtTFA) in mitochondrial dysfunction and characterizing the role of new metabolic pathways involved in the pathophysiology of FRDA^17^.

Recent studies have shown that miRNAs are involved in altered gene expression profiles that trigger the development of mitochondrial diseases and cellular redox homeostasis. In fact, miRNAs participate in the regulation of frataxin levels^18, 19^.

Small, single-stranded RNA molecules, or miRNAs, measure 18 to 22 nt in length and regulate gene expression by binding to their target mRNAs; miRNAs can be detected in many tissues and even in biological fluids such as serum, saliva or urine, where they are resistant to degradation by RNAases^19, 20^. Although a small number of studies have analysed miRNAs in FRDA^21, 22^, their regulatory role in this disease has not been clearly reported. Of the two previous studies, only one was performed on blood, reporting differential levels of miR-886 in FRDA blood samples^21^. However, miR-886 is not actually a miRNA, and it has been reclassified as a vault RNA in the most recent version of miRbase, v21 (miRNA accession number: MI0005527; available at www.mirbase.org).

The potential of identifying miRNA signatures in FRDA goes beyond the discovery of physiological and molecular pathways underlying this disease. Understanding the phenotypic variability of patients is also necessary for designing the most appropriate therapy for each of them according to their specific pattern of disease progression. Many therapies have been suggested on the basis of a single metabolic pathway datum in FRDA, without considering other molecular mechanisms underlying the disease or the clinical evolution of the patients^21^. Clarification of miRNA signatures could therefore provide a new landscape of pathological mechanisms occurring during the natural history of the disease, since miRNA levels can change with disease progression and pharmacological interventions.

In this study, we analysed the levels of circulating miRNAs in plasma from FRDA patients using small RNA sequencing. This is currently the most precise and sensitive method for mapping and quantifying RNA transcripts. In addition, we validated seven miRNA candidates using qRT-PCR, providing a new algorithm for the diagnosis of FRDA. Among these candidates, we found that miRNA-323 is a potential biomarker for monitoring cardiomyopathy progression.

## Results

### Description of participants

Twenty-five Caucasian FRDA patients from different families were enrolled in this study, all of whom had their diagnosis confirmed by genetic testing. Participants with neoplastic diseases and active infection were excluded. Thirteen patients were men (52%), and the mean age of the group was 38 years. Table 1 shows the clinical characteristics of the patients participating in the study. Eight FRDA patients (32%) had also been diagnosed with cardiomyopathy. This diagnosis did not appear in the clinical records of the remaining 17 participants at the time of the study.

**Table 1:** Demographic and clinical features.

Patients (N)	25
Sex (M/F)	13 (52%)/12 F (48%)
Age (mean/range)	39 years (19–68)
Age at onset (mean/range)	16 years (6–38)
Disease duration (mean/range)	22 years (8–35)
Expanded CAG repeats in the larger allele (mean/range)	665 (25–1185)
SARA score (average /range)	26,27 (9–37)
Diabetes (N/%)	5 (20%)
Smokers (N/%)	4 (16%)
Cardiomyopathy (N/%)	8 (32%)

This study also included 25 Caucasian healthy controls with no neoplastic diseases, active infection, cardiomyopathy, heart problems, hypertension, or diabetes. Of these healthy volunteers, 13 were men (52%), and the mean age of the group was 39 years (range: 16–56 years).

### Identification of differentially expressed miRNAs using next-generation sequencing (NGS)

We first analysed miRNA samples from 25 FRDA patients and 17 healthy controls using next-generation sequencing. A preliminary exploration of the expression levels of all miRNAs showed that patient and control samples were well separated with the exception of five patients (corresponding to P.28, P.37, P.38, P.41 and P.43, Supplementary Fig. S1).

**Figure 1:**
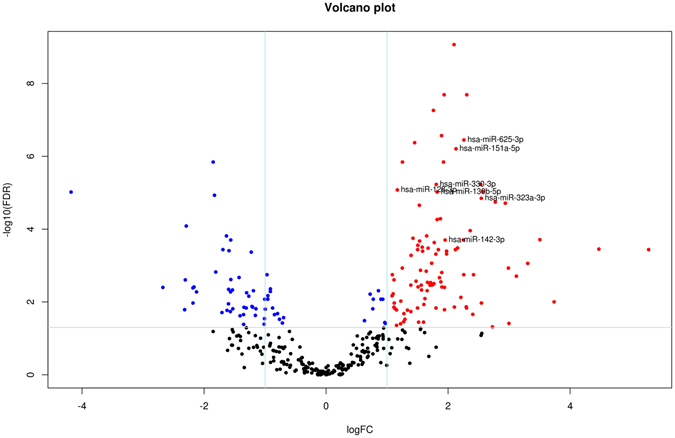
Volcano plot of differentially expressed human mature miRNAs in patients versus controls. Vertical lines indicate the threshold for a relative expression fold change (FC) of 2 or -2 fold compared to controls. The horizontal line represents the threshold of a 0.05 FDR value. The red and blue points lying in the top right and top left sectors are significantly upregulated and downregulated, respectively, in patient versus control samples (FDR < 0.05, FC ≥ 2 or ≤ −2). miRNAs selected by LASSO and cross validation are labelled.

Differential expression analysis between patients and controls showed differential expression of 164 miRNAs between the two groups (false discovery rate <0.05): 110 miRNAs were upregulated and 54 downregulated in samples from patients compared to controls. Among them we found 26 miRNAs with a false discovery rate (FDR) of less than 1e-4 and 12 with a correlation level lower than 0.7 (Supplementary Fig. S2). A LASSO logistic regression model with binomial distribution was fitted with 12 variables and 42 observations (considering all the samples). The minimum leave-one-out cross-validation error was 0.071. The miRNAs identified at the optimal value were hsa-miR-128-3p, hsa-miR-625-3p, hsa-miR-130b-5p, hsa-miR-151a-5p, hsa-miR-330-3p, hsa-miR-323a-3p, and hsa-miR-142-3p. These seven miRNAs were upregulated in patients compared to controls (Fig. 1).

### Validation of the differentially expressed miRNAs by RT-qPCR

**Figure 2:**
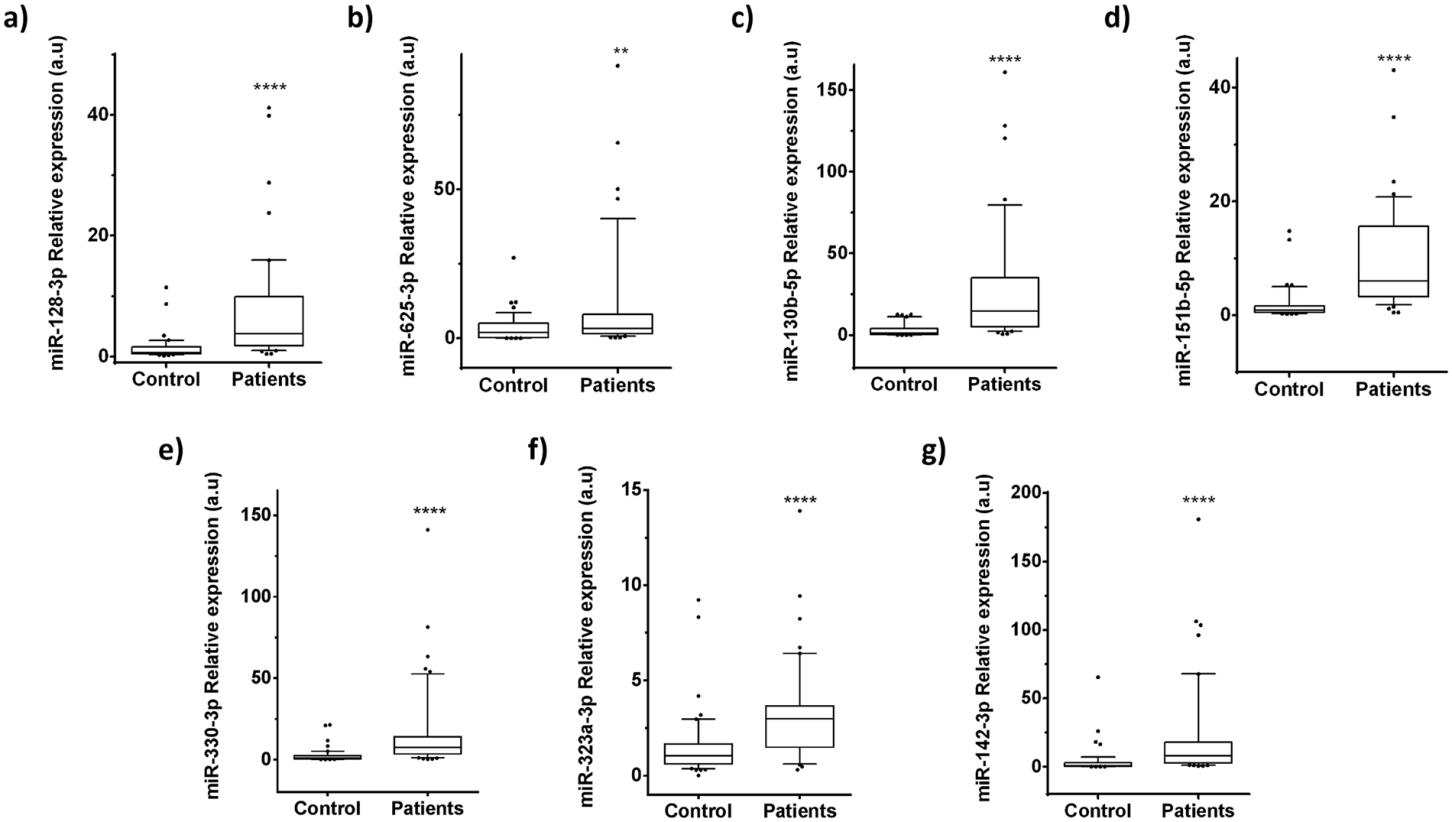
Relative expression levels of the miRNAs with different representation found in plasma of FRDA patients compared to healthy control participants. Box plot of plasma levels of (**a**) miR-128-3p (p < 0.0001); (**b**) miR-625-3p (p = 0.0264); (**c**) miR-130b-5p (p < 0.0001); (**d**) miR-151a-5p (p < 0.0001); (**e**) miR-330-3p (p < 0.0001); (**f**) miR-323a-3p (p < 0.0001); and (**g**) miR-142-3p (p < 0.0001) in healthy participants (Controls) (n = 25) and FRDA patients (n = 25). Expression levels of the miRNAs were normalized to miR-16. The lines inside the boxes denote the medians. The boxes mark the interval between the 25th and 75th percentiles. The whiskers denote the interval between the 10th and 90th percentiles. Filled circles indicate data points outside the 10th and 90th percentiles. Statistically significant differences were determined using Mann-Whitney tests. All P-values were two-tailed and less than 0.05 was considered statistically significant.

The seven differentially expressed miRNAs detected by NGS were validated by RT-qPCR (Supplementary Table S1). We analysed these miRNAs in the 25 FRDA patients and in the 25 controls (comprising 17 sequenced samples plus 8 additional controls). The two groups were matched by age, sex and race. We calculated relative expression levels for each miRNA, using hsa-mir-16-5p as an endogenous control due to its stable counts and threshold cycle (Ct) values in all the samples analysed by NGS and RT-qPCR, respectively. We detected a Ct of less than 39 in all miRNAs, with the exception of mir-130b-5p and mir-142-3p (Ct < 41). All miRNAs were present in plasma at higher levels in patients compared to healthy controls, in agreement with the results obtained by small RNA sequencing (Fig. 2). The same differences were observed when we performed the analysis taking sex and age into account (all p-values less than 0.05; Supplementary Tables S2 and S3).

Receiver operating characteristic curve (ROC) analyses performed to evaluate the diagnostic value of the seven circulating miRNAs revealed that all of them could be used to distinguish FRDA cases from healthy controls. The area under the ROC curve (AUC), standard error, 95% confidence interval (CI), fold change cut-off value, sensitivity and specificity for each miRNA are shown in Table 2. Of the seven miRNAs analysed to elucidate their potential as biomarkers for FRDA diagnosis, miR-151a-5p showed the best ROC curve parameters (AUC = 0.88, sensitivity = 92.0%, specificity = 80.0%).

**Table 2:** Area under ROC curves for FRDA diagnosis.

miRNA	AUC	Standard error	95% CI	Fold change optimal cut-off value	Sensitivity (%)	Specificity (%)
miR-128-3p	0.85	0.07	0.74, 0.96	1.50	84.0	72.7
miR-625-3p	0.69	0.08	0.53, 0.80	2.17	72.0	54.5
miR-130b-5p	0.90	0.05	0.81, 0.98	2.80	92.0	72.7
miR-151a-5p	0.88	0.05	0.78, 0.98	1.85	92.0	80.0
miR-330-3p	0.84	0.06	0.73, 0.96	2.21	84.0	72.7
miR-323a-3p	0.80	0.07	0.67, 0.94	1.48	88.0	72.7
miR-142-3p	0.85	0.06	0.73, 0.96	2.14	88.0	72.7

### Phenotypic characterization of FRDA patients according to miRNA expression

In a second approach, we analysed the expression of these seven miRNAs in plasma samples taken from the FRDA patients (n = 25). This approach was selected in order to identify specific miRNA signatures corresponding to phenotypic and clinical features of FRDA. The patients were divided into subgroups according to sex, age, and comorbidities (diabetes and cardiomyopathy). We did not observe any significant differences in miRNA expression for sex, age, and diabetes. However, we found that miR-323a-3p was significantly upregulated in patients with FRDA and cardiomyopathy (n = 8) in comparison with the remaining FRDA patients (n = 17; Table 3).

**Table 3:** Expression levels of selected miRNAs in FRDA cases.

miRNA	Cardiomyopathy		P value*
	Yes (n = 8) FC (SD)	No (n = 17) FC (SD)	
miR-128-3p	5.02 (3.49)	7.07 (9.82)	>0.05
miR-625-3p	13.72 (29.55)	29.55 (63.14)	>0.05
miR-130b-5p	28.43 (39.70)	40.49 (47.31)	>0.05
miR-151a-5p	9.38 (9.64)	9.24 (7.00)	>0.05
miR-330-3p	12.19 (17.51)	17.51 (28.10)	>0.05
miR-323a-3p	4.82 (3.52)	2.56 (1.35)	0.048
miR-142-3p	23.35 (25.14)	20.43 (36.26)	>0.05

*Statistically significant differences were determined using Mann-Whitney tests. All P-values were two-tailed and less than 0.05 was considered statistically significant.

### miR-323a-3p as a biomarker for diagnosis of cardiomyopathy in FRDA

We performed the chi-squared test to test the association between miRNA-323a-3p fold change and cardiomyopathy, we found that of the eight FRDA patients with cardiomyopathy, seven (87.5%) showed a fold change above 2.5, and of those not affected by cardiomyopathy, just 41.2% presented a fold change value above 2.5 (p = 0.048) (Table 3). In order to explore the value of this miRNA as a biomarker for cardiomyopathy, we studied the coefficient of variation (CV) for miR-323a-3p in different datasets a) 17 controls from our sequencing data study, b) 27 controls from an aortic stenosis circulating miRNA profile analysis^23^, and c) seven controls from a study on miRNA analysis in adverse prognosis of myelodysplastic syndromes (https://www.ebi.ac.uk/arrayexpress/arrays/A-GEOD-18402/?ref=E-GEOD-76775). The CVs for miR-323a-3p in each analysed dataset were 0.38, 0.34 and 0.04, respectively. While the CVs of the first two datasets were similar, dataset c) gave a markedly lower value. This could be due to the smaller sample size of the third dataset in comparison with the other two, which would logically result in lower variability in expression levels.

Taking into account the sample size of each dataset and the nature of the data (human circulating miRNAs), we concluded that the miR-323a-3p CV in our control group was acceptable for assessing possible differences in expression among patient samples.

**Figure 3:**
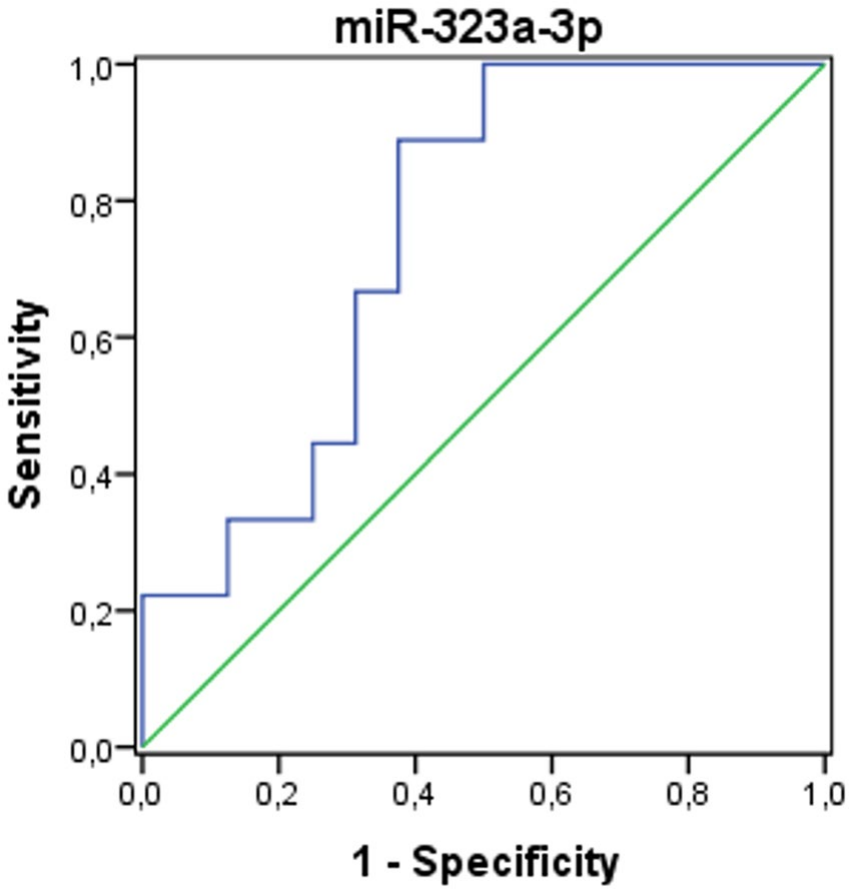
Area under curve of receiver operating characteristic (ROC curve for miR-323a-3p).

Afterwards, to examine the potential use of miR-323a-3p as a biomarker for cardiomyopathy in FRDA cases, we constructed the corresponding ROC curve, finding differences in miR-323a-3p fold change in FRDA patients with and without cardiomyopathy. We calculated the optimal cut-off value for the fold change as 2.79. Sensitivity and specificity were 88.9% and 62.5%, respectively, and the AUC was 0.75 (p = 0.042) (Fig. 3).

### Analysis of miRNA targets and pathways in the context of FRDA

In order to clarify the role of the identified miRNAs in FRDA, we analysed biochemical networks that were regulated by our seven validated miRNAs. We carried out a DIANA-miRPath v3.0 analysis and a Kyoto Encyclopaedia of Genes and Genomes (KEGG) pathway analysis to look for significantly enriched pathways. A total of 41 pathways with an FDR of less than 0.05 were retrieved in the first study. However, we focused our analysis on targets of selected miRNAs in the context of FRDA pathophysiology and identified 12 relevant pathways, which are shown in Supplementary Table S4.

Some of the most relevant pathways consist of AMPK signalling, in which genes such as FOXO1 and AMPK are directly targeted by miR-625 and miR-130b-5p. AMPK is a crosstalk protein also involved in the mTOR signalling pathway, an important mechanism that is modulated in FRDA. Our analysis found that phosphatase and tensin homolog (PTEN) was a target of miR-151-5p and miR-625-3p. Interestingly, PTEN antagonizes PI3K. As a result, the downregulation of PTEN may increase phosphatidylinositol (3, 4, 5) triphosphate (PIP3) and produce subsequent activation of AKT. Our results also demonstrate the relevance of miR-625-3p regulating the HIF-1α signalling pathway, which plays a crucial role in ATP and ROS production^24^. In addition, transferrin receptor gene (TFRC), pyruvate dehydrogenase alpha 1 (PDHA1) and lactate dehydrogenase A (LDHA) are regulated by miR-330-3p; miR-330-3p also downregulates muscle pyruvate kinase (PKM) and miR-130b-5p targets 3-phosphoinositide-dependent protein kinase 1 (PDK1) (Supplementary Fig. S3).

In our analysis, we found that miR-130b-5p regulates fatty acid synthase (FASN) and miR-142-3p targets adipose acyl-CoA synthetase-1 (ACSL1), thus regulating molecular pathways such as fatty acid metabolism and β-oxidation (Supplementary Fig. S3).

**Figure 4:**
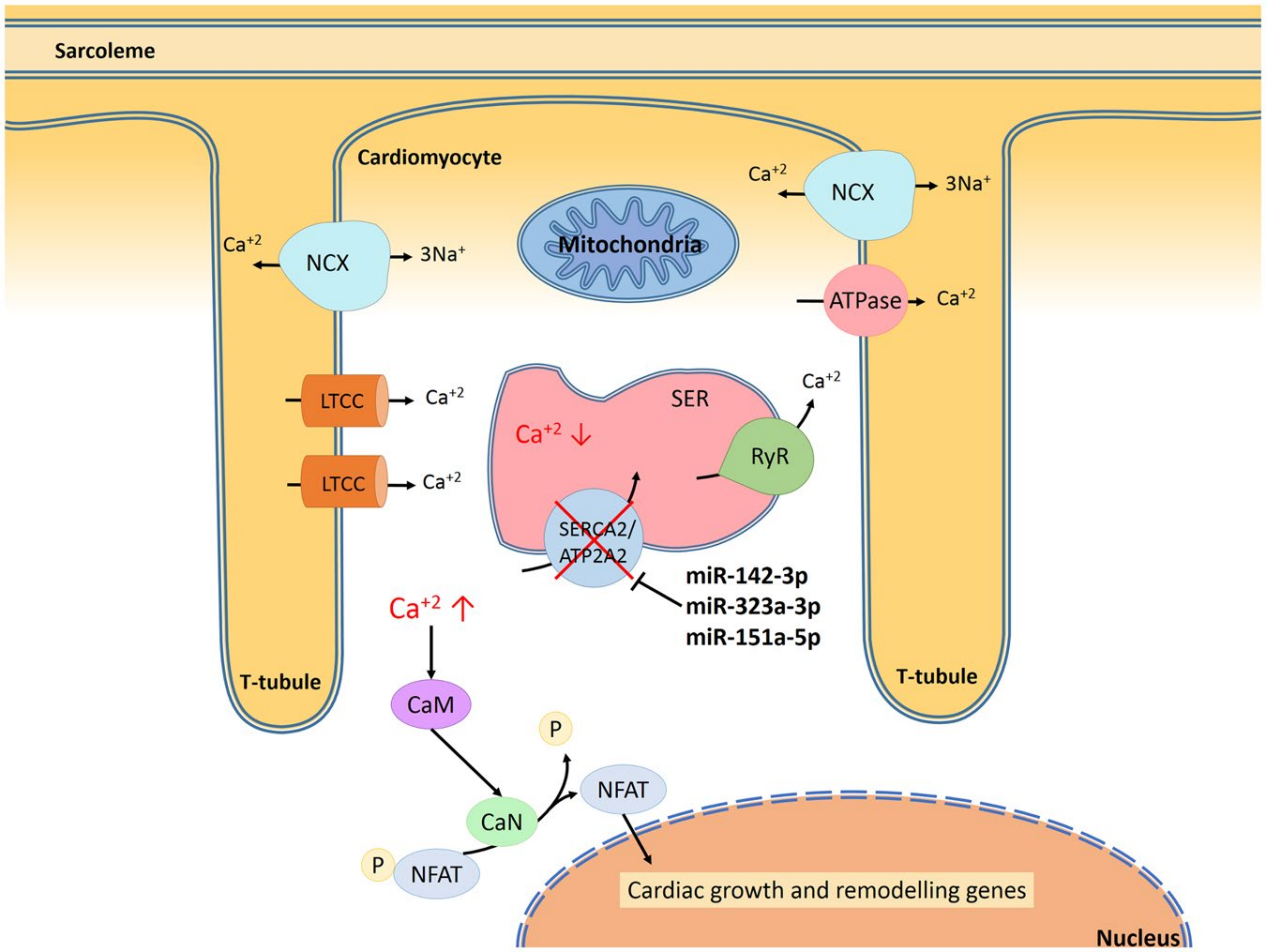
Overexpressed mir-142-3p, miR323a-3p and mir-151-5p produce cardiac hypertrophy in FRDA patients by blocking ATP2A2. Sarcoplasmic/endoplasmic reticulum Ca^+2^ ATPase (SERCA2; also known as ATP2A2) and ryanodine receptor (RyR) regulate the Ca^+2^ input and output (respectively) of the sarcoplasmic endoplasmic reticulum (SER) in cardiomyocytes. In addition, sarcolemmal Na^+^/Ca^+2^ exchangers (NCX), ATPases, mitochondria, and L-type Ca^+2^ channels (LTCC) mediate the exchange of cytosolic Ca^+2^. Increased levels of mir-142-3p, miR323a-3p and mir-151-5p may decrease mRNA levels of SERCA and also SERCA protein levels. Low levels of SERCA produce an elevated cytosolic level of Ca^2+^ with a concomitant activation of calmodulin protein (CaM). Activated CaM can, in turn, stimulate the active form of calcineurin (CaN), which dephosphorylates the nuclear factor of activated T cells (NFAT) protein in the cytosol. This facilitates its translocation into the nucleus, and interacts with cardiac growth and gene promoter remodeling, resulting in increased cardiac hypertrophy.

Other pathway analyses revealed that the insulin-signalling pathway is also targeted by some miRNA candidates. In fact, glycogen synthase kinase 3 beta (GSK3β) is regulated by both miR-130b-5p and miR-625-3p. Finally, Wnt/β-catenin signalling related genes such as catenin (CTNNB1) and calcium-transporting ATPase sarcoplasmic reticulum type (ATP2A) are targets of some miRNAs: CTNNB1 is targeted by miR-151-5p and miR-625-3p and ATP2A2 is targeted by miR-142-3p, miR-323a-3p, and miR-151a5p. Both CTNNB1 and ATP2A are relevant genes found in hypertrophic hearts. We hypothesize that an elevated cytosolic level of Ca^+2^ due to low levels of ATP2A2 (SERCA) may stimulate calmodulin protein (CaM), and in turn activate calcineurin (CaN), which dephosphorylates the nuclear factor of activated T cells (NFAT) being translocated into the nucleus. This transcription factor activates genes for cardiac growth and remodelling, resulting in increased cardiac hypertrophy (Fig. 4).

### Validation of pathway in FRDA neuroblastoma cell line model

**Figure 5:**
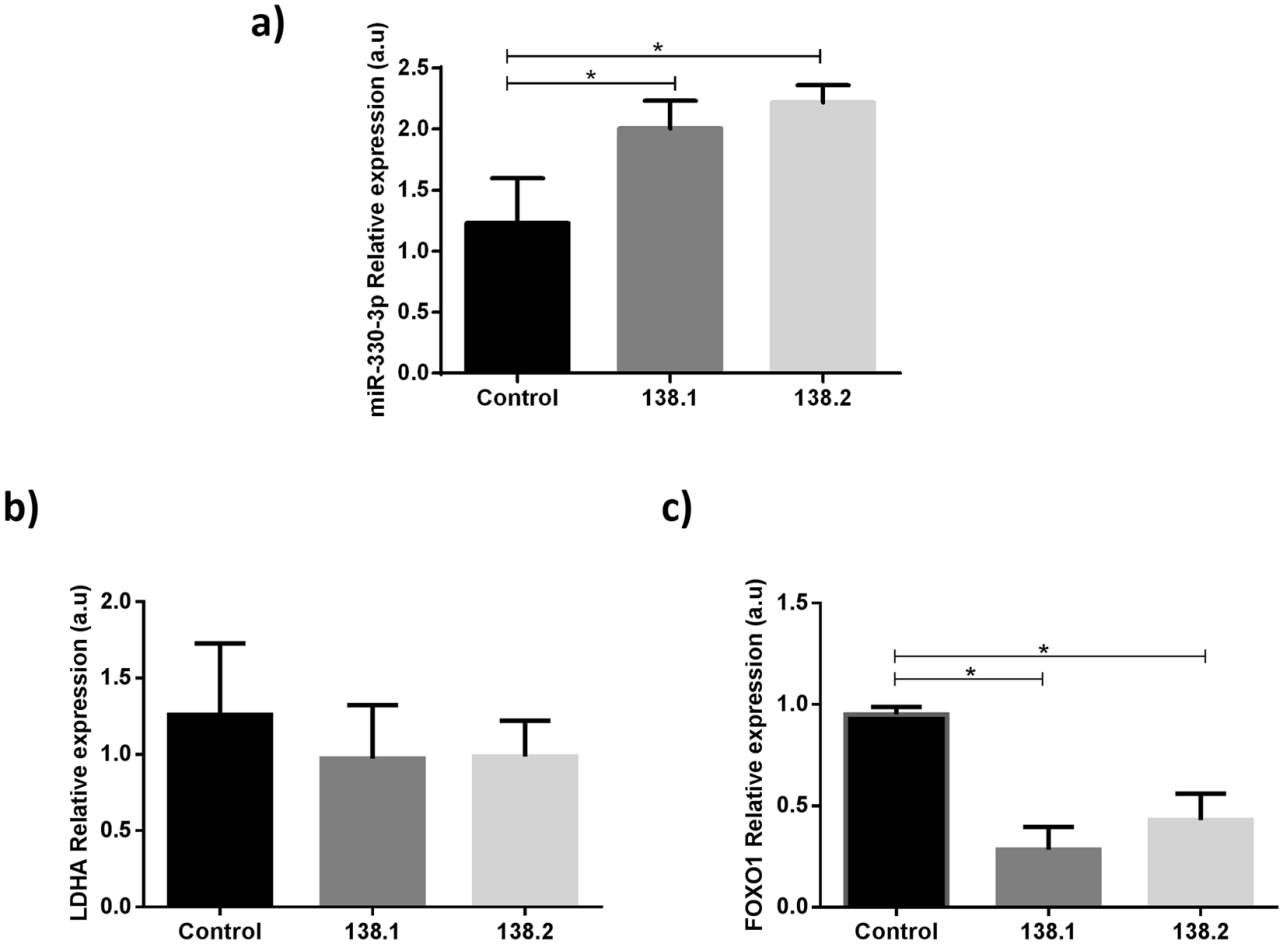
Relative expression levels in control SH-SY5Y and clones FXN-138.1 and FXN-138.2. (**a**) miR-330-3p relative expression levels (p < 0.01); (**b**) LDHA relative expression levels; and (**c**) FOXO1 relative expression levels (p < 0.05). Expression levels of the miRNA were normalized to RNU48 and gene expression was normalized to GAPDH. Statistically significant differences were determined using Mann-Whitney tests. Results are means ± SD (n = 3). All P-values were two-tailed and less than 0.05 was considered statistically significant.

In order to identify the relevance of some miRNAs as potential contributors in FRDA disease mechanisms, we tested their expression in shRNA FXN-silenced SH-SY5Y models (138.1 and 138.2 FXN-silenced lines). We found that mir-330-3p was overexpressed in silenced clones compared to the control cell line (Fig. 5a); however, we did not observe differences in other miRNAs (data not shown). Interestingly, miRpath identified miR-330-3p as having one of the largest numbers of gene targets in the selected pathways (Supplementary Fig. S3). For this reason, we analysed the gene expression of two targets of mir-330-3p, LDHA and FOXO1. LDHA mRNA levels showed a tendency to be downregulated in FXN-silenced cell lines compared to the control (Fig. 5b), but FOXO1 mRNA levels were downregulated in 138.1 and 138.2 FXN-silenced lines (Fig. 5c).

## Discussion

We studied the circulating miRNome in FRDA patients and examined how miRNAs can contribute to the characterization of new molecular and epigenetic regulatory mechanisms participating in the natural history of this neuromuscular disease. Indeed, miRNAs are promising molecules that can be used as biomarkers for the diagnosis, prognosis, and treatment monitoring of FRDA. This means that we could use miRNAs to screen and consolidate therapies, improving the quality of life of FRDA patients and reducing costs in clinical trials through the design of more personalized treatments and the identification of different molecular pathways underlying the progression of the disease.

The role of miRNAs is attracting significant interest in the area of neuromuscular disorders^25^ and cardiovascular diseases^26^. This is the first report of a comprehensive investigation of circulating miRNAs as biomarkers of FRDA. In our study, through small RNA sequencing, we found seven circulating miRNAs (hsa-miR-128-3p, hsa-miR-625-3p, hsa-miR-130b-5p, hsa-miR-151a-5p, hsa-miR-330-3p, hsa-miR-323a-3p, hsa-miR-142-3p) differentially represented in plasma from FRDA patients versus healthy controls. Some of these circulating miRNAs have already been found in other neurodegenerative disorders. For example, miR-128-3p was found overexpressed in plasma from people with Huntington’s disease^27^ and in T cells from people with multiple sclerosis^28^. In contrast, lower levels of miR-142-3p were found in plasma of patients with Alzheimer’s disease than in controls^29^.

In an effort to identify biomarkers to stratify patients’ risk according to their phenotype, we found that miR-323a-3p was significantly upregulated in patients with cardiomyopathy compared to patients whose clinical records made no mention of this comorbidity. Our analysis demonstrated high sensitivity (88.9%) and acceptable specificity (62.5%). The AUC was 0.75 (p = 0.042).

From a clinical point of view, the Mitochondrial Protection with Idebenone in Cardiac or Neurological Outcome (MICONOS) study group concludes that, irrespective of neurological status, all FRDA patients need an initial cardiac evaluation including cMRI (cardiac magnetic resonance imaging) and echocardiography and a regular echocardiographic follow-up^30^. Furthermore, these authors assert that clinical algorithms must be developed to manage and predict cardiomyopathies in rare diseases. The use of miR-323a-3p would help clinicians in this regard and could contribute to early diagnosis and prognosis of cardiomyopathy prior to detection by standard diagnostic procedures of clinical or morphological cardiac tissue manifestations.

One of the pathways identified in our study is the AMPK signalling pathway, which is known to be altered in Friedreich’s ataxia. This pathway is responsible for altered ATP levels and deregulation of the mitochondrial biogenesis pathway, as previously described^17^. AMPK, which is directly targeted by miR-625-3p and miR-130b-5p, is a crosstalk protein involved in the mTOR signalling pathway, an important mechanism that is altered in FRDA^17^. Calap-Quintana *et al*. discovered that inhibition of TORC1 by the drug rapamycin improves the motor phenotype in *Drosophila melanogaster* FRDA models^31^. We found that miR-625-3p and miR-330-3p regulate transcription factors that play an important role in antioxidant gene regulation, protein quality control and autophagy. These transcription factors include FOXO^32–34^, one of the pathways identified in our analysis. Ataxia telangiectasia mutated (ATM), among other proteins, is also regulated by miRNAs in the FOXO pathway. ATM participates in the regulation of DNA repair and controls DNA damage responses. Altered expression levels of ATM and p53 were previously detected in lymphocytes from FRDA patients^35^. FOXO transcription factors are involved in several physiological and pathological processes, including neurological diseases^34^. To validate some of our miRNAs, we studied their expression in FXN-silenced clones of the human neuroblastoma SH-SY5Y cell line. We found that miR-330-3p was overexpressed in 138.1 and 138.2 FXN-silenced lines and FOXO1 mRNA was downregulated as a result. It will be interesting to tease out in future research the role of miR-330-3p/FOXO interplay in the regulation of neuronal survival in FRDA.

Our analysis also found that PTEN was a target of miR-151-5p and miR-625-3p. Interestingly, PTEN antagonizes PI3K, meaning the downregulation of PTEN may change the PIP3/PIP2 ratio. An increase in PIP3 could subsequently activate AKT. This pathway coordinates different steps of axon growth during development and in injury-induced axon regeneration^36^. PIP2 hydrolysis is responsible for the changes in Ca^+2^ homeostasis. Bolinches-Amorós *et al*. showed that a decrease in frataxin levels induces mitochondrial dysfunction as a result of a bioenergetic deficit and abnormal Ca^+2^ metabolism^37^. Importantly, the Ca^+2^ signalling pathway regulates many pathways. Alterations in this pathway contribute to different disease states. For this reason, the discovery of biological modulators, like miRNAs, in this pathway is highly valuable.

Some miRNAs identified in our study regulate key genes in the insulin signalling pathway, such as the eukariotic translation initiation factor eIF4E, which is regulated by miR-142-3p, and Glycogen synthase kinase 3 beta (GSK3β), which is regulated by both miR-130b-5p and miR-625-3p. In addition, insulin-like growth factor I (IGF-I) has shown therapeutic effects in different cerebellar ataxias due to its protective effects on mitochondrial function and neuroprotective effects in frataxin deficient neuronal cultures^38^ and Fxn-deficient mice^39^. Furthermore, diabetes is a metabolic disorder that affects one in every three Friedreich’s ataxia patients. Insulin resistance and loss of glucose tolerance are features found in the FRDA phenotype^40^, and they seem to depend on the entry in senescence of the islets of Langerhans^41^ and β-pancreatic cell survival.

In our pathway analysis we also identified fatty acid oxidation and central carbon metabolism, two more pathways altered in FRDA. As these results show, lactate dehydrogenase A (LDHA) deregulation may alter the NAD+/NADH ratio, thus deregulating energy metabolism through alteration of fatty acid oxidation^42^ and the Krebs cycle^43^ in cells. This deregulation of energy metabolism is a critical factor in FRDA cardiomyopathy. Linking these ideas with those concerning fatty acid pathway regulation based on our miRpath analysis, we found that miR-130b-5p regulates fatty acid synthase (FASN) and miR-142-3p targets adipose acyl-CoA synthetase-1 (ACSL1), resulting in increased uptake of fatty acids for use in β-oxidation. In addition, a prolonged energy shift from fatty acid to glucose oxidation is a well-known feature of cardiac damage^44^, and may contribute to FRDA cardiomyopathy.

Finally, we found that miR-151-5p and miR-625-3p target CTNNB1, and miR-142-3p, miR-323a-3p, and miR-151a-5p target ATP2A2. Recent studies in conditional gain-of-function of β-catenin (CTNNB1) cardiac endothelial cells from mice showed that Wnt/β-catenin signalling activation may be a cause of cardiac dysfunction through downregulation of the neuregulin-Erb-B pathway^45^. Reduced ATP2A2 mRNA levels have also been shown to affect heart function and are associated with hypertrophied hearts^46, 47^. With this study we therefore provide new candidate mechanisms to explain altered heart function in FRDA.

Our results open new avenues for developing more personalized therapies focused on specific patients’ symptoms. We identified seven miRNAs, all of which are associated with key molecular mechanisms underlying FRDA physiopathology. We found that miR-323-3p is a candidate for diagnosing cardiomyopathy in FRDA patients. Pilbrow *et al*. have described miR-323-3p as a candidate biomarker for coronary artery disease (CAD) in acute coronary syndrome (ACS) patients^48^.

Previous studies have proposed that miRNAs play a direct or indirect role in cardiac hypertrophy in FRDA. In this regard, Kelly *et al*. found that miR-155 downregulates AGTR1, resulting in reduced production of AGTR1. However, the rs5186 C allele interrupts complementarity between miR-155 and the regulatory target site of AGTR1, thereby increasing AGTR1 levels, which may explain an increased degree of cardiac hypertrophy, oxidative stress, and fibrosis in FRDA patients^49^. We explored the expression of miR-155 in our series of patients and controls but found no significant differences. This could be because, as Kelly *et al*. propose, the effect may remain in the rs5186 C allele of AGTR1, but not in the different expression levels of miR-155.

The design of this study has certain limitations that are commonly encountered in the study of rare genetic diseases. Although this is a relatively large and well-characterized FRDA cohort in comparison with previous reports, the small sample size of each sub-population limits the statistical power of the study to propose specific miRNAs that correlate with some of the clinical features analysed. Nevertheless, we are convinced that our study further increases the knowledge of molecular mechanisms underlying such a complex disease and constitutes a good starting point for launching a wider international effort to provide further insight. In any case, the potential use of these miRNAs must be seen as an additional help for clinicians and not as the main diagnostic tool. With the information provided by these miRNAs, clinicians can maintain a close follow-up of patients who show changes in the miRNA’s signature.

Both clinicians and patients have called for new drugs to treat FRDA. However, these drugs do not always provide the benefits, probably because underlying molecular mechanisms are not fully understood^50^. For example, variation in the efficacy of Idebenone in half of FRDA patients remains unexplained^51^. This drug decreases free fatty acid content^52^. Furthermore, as previously described, it decreases PGC1α^17^, which cooperates with peroxisome proliferator-activated receptor alpha (PPARα) in transcriptional control of mitochondrial fatty acid oxidation enzymes^53^. As a result, altered regulation of fatty acid metabolism and fatty acid oxidation by microRNAs may help to regulate patients’ different responses to Idebenone treatment.

In this regard, circulating miRNAs can detect pathological events, and could also monitor molecular signals participating in cardiomyopathy even before the appearance of clinical cardiac manifestations. To maximize the likelihood of detecting the onset or progression of cardiomyopathy, we suggest combining standard cardiac diagnostic procedures with the use of circulating microRNAs. This approach could provide more clinical information for evaluating cardiomyopathy progression in FRDA. In summary, miRNAs obtained in this study show new candidates for personalized therapy in FRDA patients.

## Material and Methods

### Study design and population

The study population included patients from different families who had been diagnosed with FRDA. This diagnosis was confirmed by genetic study. Patients with neoplastic diseases and active infection were excluded. Data about age, sex, tobacco use, history of diabetes, cardiopathy, medication and therapies and number of repetitions in the mutation and disease duration were recorded. The scale for assessment and rating of ataxia (SARA) was used to measure the clinical severity of the disease^54^. FRDA patients were enrolled in the study following study approval by the Biomedical Research Ethics Committee (CEIB) of Hospital La Paz (Madrid). The samples were used to create a public sample repository of FRDA in the CIBERER Biobank (www.ciberer-biobank.es; Spanish Biobank Registry number: 000161X02).

Healthy volunteers with no neoplastic diseases, active infection, cardiomyopathy, heart problems, hypertension, or diabetes were enrolled by the Basque Biobank for Research-OEHUN (www.biobancovasco.org) and the Biobank for Biomedical Research and Public Health of the Valencian Community (IBSP-CV) through the Spanish National Biobank Network (RNBB 2013/12). The participants of both groups (healthy volunteers and FRDA patients) were matched by race, sex and age (Supplementary Table S5) and were processed in the same way.

The selection process and all experimental methods were carried out in accordance with the relevant clinical guidelines, following standard operation procedures, and with the approval of the ethics and scientific committees. All experimental protocols were approved by the Biomedical Research Ethics Committee (CEIB) of Hospital La Paz (Madrid) and the ethics and scientific committees of the IBSP-CV. Informed consent was obtained from all participants.

### Cell culture

SH-SY5Y and FXN-silenced clones were grown as described in Bolinches-Amorós *et al*.^37^, and were kindly provided by Dr. Francesc Palau laboratory.

### RNA extraction and quantification

Blood samples were collected from FRDA patients and healthy participants in EDTA tubes. Each sample was centrifuged at 2500 rpm for 10 minutes to separate the plasma, then stored at −80 °C until RNA extraction. We isolated cell-free total RNA (including miRNAs) from 500 µL of plasma using the miRNeasy Serum/Plasma kit (Qiagen, Valencia, CA, USA), following the manufacturer’s protocol. The RNA was eluted with 25 µL of RNAse-free water. Total RNA (including miRNAs) was isolated from SH-SY5Y and FXN-silenced cell lines using the mirVana miRNA Isolation Kit (Ambion, Thermo-Fisher, Wilmington, USA), following the manufacturer’s protocol. The concentration of cell-free total RNA (including miRNAs) was quantified using NanoDrop ND 2000 UV-spectrophotometer (Thermo Scientific, Wilmington, DE, USA).

### Circulating miRNA analysis

In order to analyse the circulating miRNAs obtained from FRDA patients and healthy controls, we first performed Next Generation Sequencing analysis and then validated miRNA levels with differential expression obtained from the previous step by RT-qPCR.

### Library preparation and next-generation sequencing

Small-RNA libraries were prepared using the NEBNext Multiplex Small RNA Library Prep Set for Illumina (Set 1&2) (New England Biolabs, MA, USA), following the manufacturer’s protocol. Briefly, 5′ and 3′ adapters were ligated with small RNA samples, followed by a cDNA library construction and incorporation of index tags by reverse transcription-PCR (RT-PCR). The products of this RT-PCR were purified using 6% non-denaturing polyacrylamide gel electrophoresis, and a 145-160 bp size fraction was isolated. The cDNA library samples were used for cluster generation and Illumina sequencing on the HiScanSQ platform (50 bp single read).

The first step was to assess the quality of the Illumina raw sequences with the FastQC software. Based on the results obtained, the sequence reads were trimmed to remove sequencing adapters and low quality bases. Once the data were deemed of sufficient quality, they were mapped against the human Hg38 build reference sequence, taken from the UCSC Genome Browser. After that, the intersection between the aligned position of reads and the miRNA coordinates taken from miRBase v21 was performed. The alignment and quantification steps were performed using the Subread^55^ and RSubread^56^ packages, respectively.

### Differential expression analysis

The expression data were normalized using the trimmed mean of M-values (TMM) method^57^. Differential expression analysis was performed between patients and controls. The test used was based on exact statistical methods developed by Robinson and Smyth^58^. It was necessary to estimate miRNA-specific dispersion with a quantile-adjusted conditional maximum likelihood (qCML) method^58, 59^. Dysregulated miRNAs with an FDR of less than 1e-4 were used to calculate a correlation matrix. A logistic regression model with a LASSO penalty^60^ was fitted with miRNAs that had a correlation level below 0.7. In order to select the most important miRNAs in the model, a leave-one-out cross validation was performed. All miRNAs that had non-zero coefficients at the value of λ that gave the minimum mean cross-validated error were selected.

### Prediction of miRNA targets and over-representation analysis

All miRNAs have a large number of potential target sites. The computational approach to predicting miRNA targets helps to narrow down the potential candidates. In our approach, we first used DIANA-microT-CDS accessed from DIANA web server v5.0^61^. This tool shows whether the target was also predicted by miRanda or TargetScan or was experimentally validated in TarBase v7.0. We used the DIANA-miRPath v3.0 functional analysis online suite to identify miRNAs controlling significant molecular pathways annotated on Kyoto Encyclopaedia of Genes and Genomes (KEGG), using as default parameters: experimentally supported interactions from DIANA TarBase v.7.0; a p-value threshold of 0.001; and a microT threshold of 0.8. TargetScan was used to predict targets for hsa-miR-128-3p. To reduce the number of false positive miRNA targets, we applied a false discovery rate (FDR) correction to selected KEGG pathways. The algorithm used in this analysis was a one-tailed Fisher’s exact test^62^.

### Real-time qPCR validation of a novel miRNA signature from plasma of FRDA patients and healthy controls

Reverse transcription reactions were performed using the TaqMan miRNA Reverse Transcription kit and miRNA-specific stem-loop primers (Part No. 4366597, Applied Biosystems. Inc, CA; USA) and 100 ng of input cell-free RNA in a 20 µL RT reaction. Real-time PCR reactions were performed in triplicate, in scaled-down 10 µL reaction volumes using 5 µL TaqMan 2x Universal PCR Master Mix (Applied Biosystems. Inc, CA; USA) with No UNG, 0.5 µL TaqMan Small RNA assay (20x) (Applied Biosystems. Inc, CA; USA) [hsa-miR-128-3p (002216), hsa-miR-625-3p (002432), hsa-miR-130b-5p (002114), hsa-miR-151a-5p (002642), hsa-miR-330-3p (000544), hsa-miR-323a-3p (002227), hsa-miR-142-3p (000464), hsa-miR-16-5p (000391)], 3.5 µL of nuclease free water and 1 µL of RT product. Real-time PCR was carried out on an Applied BioSystems 7900HT thermocycler (Applied Biosystems. Inc, CA; USA) programmed as follows: 50 °C for 2 minutes, 95 °C for 10 minutes followed by 45 cycles of 95 °C for 15 seconds and 60 °C for 1 minute. We used hsa-miR-16-5p (000391), one of the most stable miRNAs in terms of read counts, and which has been used previously as an endogenous control^63^, to normalize the expression in plasma samples. RNU48 (001006), meanwhile, was used to normalize the expression in cell-line samples. All the fold-change data were obtained using the delta-delta CT method (2^−ΔΔCT^)^64^.

### Validation of miRNAs as biomarkers

To assess differences between patients and healthy controls and between different patient subgroups, we performed several statistical tests, using patient phenotype, age, sex, and disease onset as variables. Student’s T tests and Mann-Witney tests were applied to compare miRNA fold-change values as a continuous variable in the different groups of study participants (healthy controls, FRDA patients with metabolic disorders and FRDA patients without metabolic disorders). Chi-square tests were employed to compare frequencies between the study groups when the fold-change was converted into a categorical variable (less than 2.5, greater than 2.5).

The coefficient of variation of miR-323a-3p was calculated by dividing the standard deviation by the mean. Variance stabilization was performed on our sequencing data before the coefficient of variation was calculated, as RNA-Seq data tend to follow the Negative Beta Binomial model, and so their SD tends to increase with mean. The data from the microarray datasets, meanwhile, were normalized using the RMA method^65^.

The miRNA diagnostic test from each miRNA was validated by ROC curves analysis: area under the curve, diagnostic sensitivity and specificity, positive and negative predictive values. Optimal cut-off points were determined by highest sensitivity plus specificity and efficiency values.

P-values less than 0.05 were considered statistically significant. The data analysis was performed using SPSS version 20 (IBM Corporation).

### Gene expression of mir-330-3p targets

Reverse transcription reactions were performed using the High-Capacity cDNA Reverse Transcription Kit and 200 ng of input cell-free RNA in a 20 µL RT reaction.

Real-time PCR reactions were performed in duplicate, in scaled-down 10 µL reaction volumes using 5 µL 2x TaqMan Universal PCR Master Mix (Applied Biosystems. Inc, CA; USA) and 0.5 µL TaqMan® Gene Expression Assays (20x) [LDHA gene (Hs01378790_g1); FOXO1 gene (Hs00231106_m1)]. Real-time PCR was carried out on an Applied BioSystems 7900HT thermocycler (Applied Biosystems. Inc, CA; USA), programmed as follows: 50 °C for 2 minutes, 95 °C for 10 minutes followed by 40 cycles of 95 °C for 15 seconds and 60 °C for 1 minute. The GAPDH gene (Hs02786624_g1) was used to normalize the expression data, using the delta-delta CT method (2^−ΔΔCT^)^64^.

## Electronic supplementary material

Supplementary Information
