# Supplementary material for: Circulating miR-323-3p is a biomarker for cardiomyopathy and an indicator of phenotypic variability in Friedreich’s ataxia patients
Source: Sci Rep. 2017 Jul 12;7:5237. doi: 10.1038/s41598-017-04996-9 (PMC5507909; doi:10.1038/s41598-017-04996-9)
Supplement: Supplementary file 1 — Supplementary Information [file 41598_2017_4996_MOESM1_ESM.pdf]

Supplementary Information

Title: Circulating miR-323-3p is a biomarker for cardiomyopathy and an indicator of phenotypic variability in Friedreich's ataxia patients.

Authors:

Seco-Cervera M.,[1,2,3] González-Rodríguez D.,[2] Ibáñez-Cabellos, J.S.,[1,2,3] Peiró-Chova, L.,[2] González-Cabo P.,[1, 3] García-López E.,[3] Vilchez J.J.,[1, 4] Sanz-Gallego I.,[5] Pallardó F.V. [1, 2, 3,*], García-Giménez J.L.,[1, 2, 3, *]

Supplementary Table S1. miRNAs selected as biomarkers for FRDA.

miRNA name	Mature sequence	Accession
**hsa-miR-128-3p**	**ucacagugaaccggucucuuu**	**MIMAT0000424**
**hsa-miR-625-3p**	**gacuauagaacuuucccccuca**	**MIMAT0004808**
**hsa-miR-130b-5p**	**acucuuucccuguugcacuac**	**MIMAT0004680**
**hsa-miR-151a-5p**	**ucgaggagcucacagucuagu**	**MIMAT0004697**
**hsa-miR-330-3p**	**gcaaagcacacggccugcagaga**	**MIMAT0000751**
**hsa-miR-323a-3p**	**cacauuacacggucgaccucu**	**MIMAT0000755**
**hsa-miR-142-3p**	**uguaguguuuccuacuuuaugga**	**MIMAT0000434**
**hsa-miR-16-5p**	**uagcagcacguaaauauuggcg**	**MIMAT0000069**

Supplementary Table S2. Expression levels of selected miRNAs in FRDA patients and controls stratified by age.

miRNA	<39 years			>39 years		
	Patients (n=14) FC (SD)	Controls (n=11) FC (SD)	P value*	Patients (n=11) FC (SD)	Controls (n=14) FC (SD)	P value*
miR.128-3p	4.24 (4.56)	1.63 (2.41)	0.021	9.18 (11.10)	1.04 (0.75)	< 0.0001
miR-625-3p	13.46 (21.05)	1.48 (2.02)	0.007	20.38 (30.24)	2.72 (2.10)	0.034
miR-130b-5p	33.66 (50.49)	4.08 (4.67)	0.039	40.41 (37.69)	1.87 (2.18)	< 0.0001
miR-151a-5p	7.03 (6.72)	2.06 (4.00)	0.002	12.16 (10.40)	1.58 (1.57)	< 0.0001
miR-330-3p	11.73 (18.15)	3.03 (6.11)	0.021	21.00 (30.79)	1.93 (2.29)	< 0.0001
miR-323a-3p	2.84 (2.00)	1.63 (2.41)	0.049	3.83 (2.78)	1.30 (0.92)	< 0.0001
miR-142-3p	18,64 (26.50)	5.35 (13.46)	0.008	24.83 (40.17)	2.54 (3.85)	< 0.0001

*Statistically significant differences were determined using Mann Whitney tests. All P-values were two-tailed and less than 0.05 was considered statistically significant.

Supplementary Table S3. Expression levels of selected miRNAs in FRDA patients and controls stratified by sex.

miRNA	Males			Females		
	Patients (n=12) FC (SD)	Controls (n=13) FC (SD)	P value*	Patients (n=13) FC (SD)	Controls (n=12) FC (SD)	P value*
miR.128-3p	8.02 (11.45)	1.08 (0.64)	0.001	4.93 (3.54)	1.54 (2.63)	0.002
miR-625-3p	14.47 (23.20)	2.44 (1.96)	0.05	17.80 (26.94)	1.67 (2.28)	0.007
miR-130b-5p	49.27 (57.21)	3.39 (3.40)	< 0.0001	24.96 (25.72)	2.17 (3.65)	< 0.0001
miR-151a-5p	11.14 (11.17)	1.18 (0.78)	< 0.0001	7.57 (5.63)	2.46 (4.00)	0.005
miR-330-3p	22.59 (33.66)	1.49 (1.05)	< 0.0001	9.55 (8.00)	3.43 (6.12)	0.011
miR-323a-3p	2.62 (1.20)	1.26 (0.72)	0.004	3.89 (3.02)	1.80 (2.40)	0.016
miR-142-3p	28.82 (44.32)	1.47 (1.34)	< 0.0001	14.48 (14.77)	6.29 (13.10)	0.008

*Statistically significant differences were determined using Mann Whitney tests. All P-values were two-tailed and less than 0.05 was considered statistically significant.

Supplementary Table S4. KEGG pathways targeted by differential expressed miRNAs in FRDA.

KEGG pathway	KEGG CODE
**Fatty acid biosynthesis**	**hsa00061**
**Lysine degradation**	**hsa00310**
**Arrhythmogenic right ventricular cardiomyopathy (ARVC)**	**hsa05412**
**Central carbon metabolism in cancer**	**hsa05230**
**AMPK signalling pathway**	**hsa04152**
**mTOR signalling pathway**	**hsa04150**
**Ubiquitin mediated proteolysis**	**hsa04120**
**FoxO signalling pathway**	**hsa04068**
**Fatty acid metabolism**	**hsa01212**
**HIF-1 signalling pathway**	**hsa04066**
**Insulin signalling pathway**	**hsa04910**
**Signalling pathways regulating pluripotency of stem cells**	**hsa04550**

Supplementary Table S5. List of patients and controls matched by gender and age.

PATIENTS			*CONTROLS*		
**ID**	**GENDER**	**AGE**	**ID**	**GENDER**	**AGE**
27	FEMALE	26	33	FEMALE	24
38	FEMALE	32	34	FEMALE	33
42	FEMALE	68	49	FEMALE	56
41	FEMALE	48	46	FEMALE	53
2	FEMALE	39	47	FEMALE	38
6	FEMALE	56	47	FEMALE	56
18	FEMALE	38	47	FEMALE	38
5	FEMALE	46	31	FEMALE	44
25	FEMALE	46	31	FEMALE	44
29	MALE	28	35	MALE	30
1	MALE	34	10	MALE	32
4	MALE	35	10	MALE	32
13	MALE	35	10	MALE	32
30	MALE	32	10	MALE	32
14	MALE	41	22	MALE	40
39	FEMALE	49	7	FEMALE	54
17	MALE	47	20	MALE	47
15	MALE	37	21	MALE	39
16	MALE	39	21	MALE	39
3	MALE	52	9	MALE	51
26	FEMALE	37	11	FEMALE	37
40	FEMALE	37	11	FEMALE	37
43	MALE	21	50	MALE	20
37	MALE	19	44	MALE	16
28	FEMALE	29	45	FEMALE	31

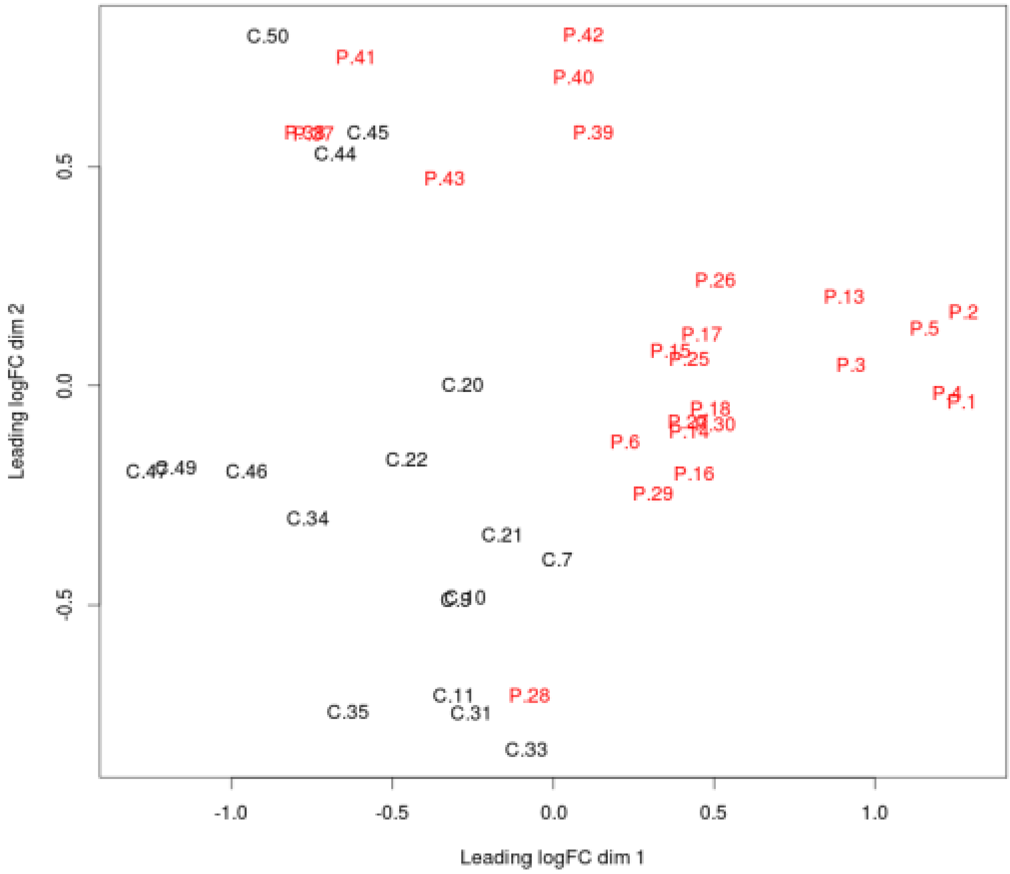

Supplementary Fig. S1. Multidimensional scale plot with all miRNAs and samples. The first component separates most of the patients from the controls. 5 patients were misclassified in this model (P.28, P.37, P.38, P.41 and P.43).

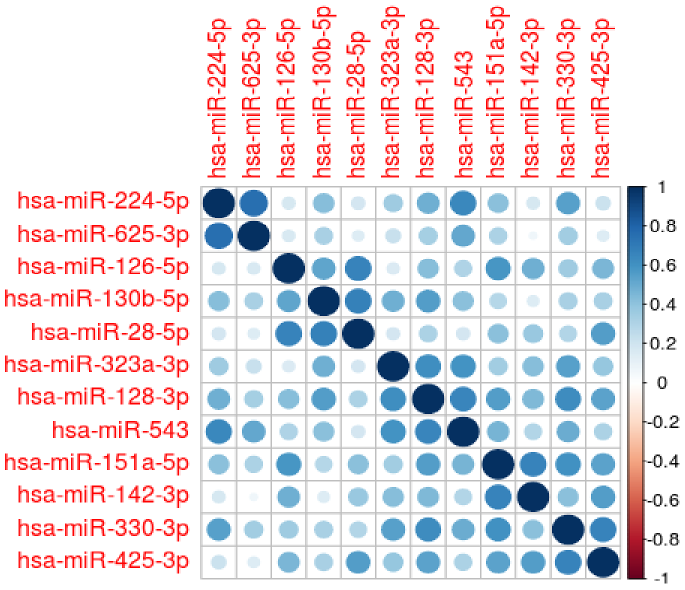

Supplementary Fig. S2. Correlation matrix. The miRNAs represented are those that had a correlation level lower than 0.7 among the most significant (FDR < 1e-4).

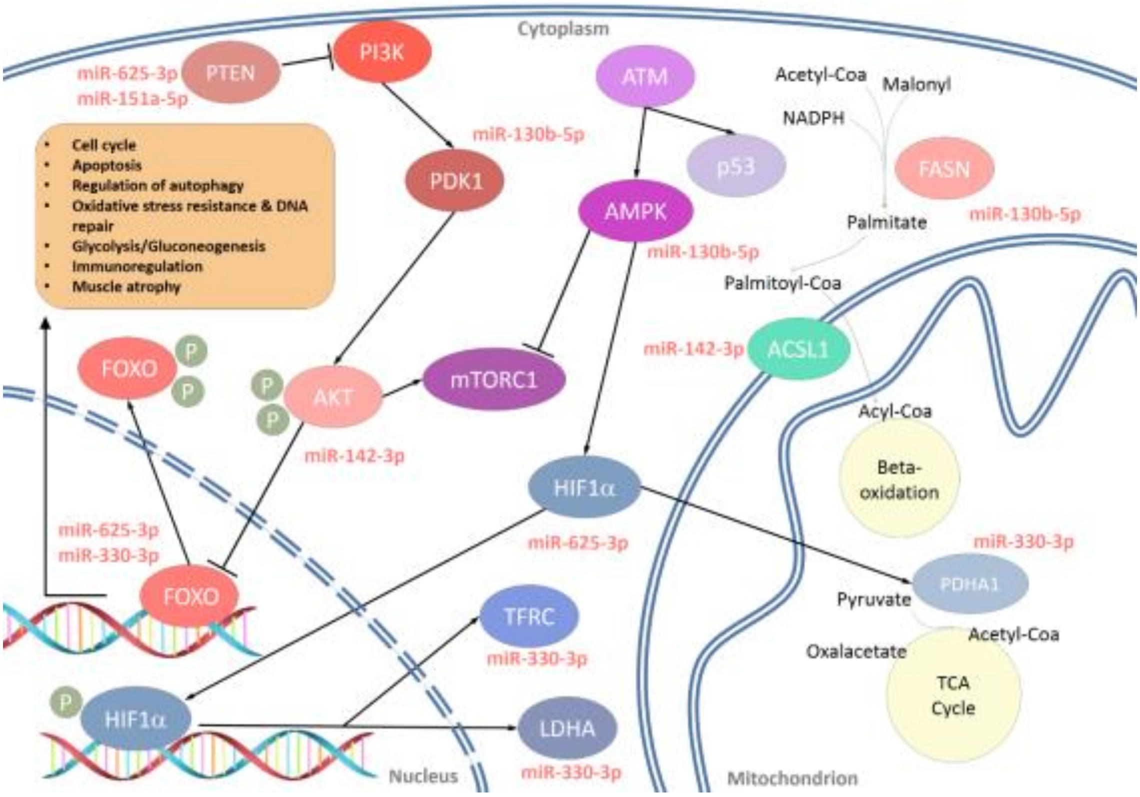

Supplementary Fig. S3. Overexpression of miRNAs in plasmas from FRDA patients, downregulate their target mRNAs. Phosphatase and tensin homolog (PTEN), Phosphatidylinositol-4,5-bisphosphate 3-kinase (PI3K), 3-phosphoinositide-dependent protein kinase 1 (PDK1), Protein kinase B (AKT), Forkhead box (FOXO), mammalian target of rapamycin complex 1 (mTORC1), Hypoxia-inducible factor 1-alpha (HIF1α), Transferrin receptor (TFRC), L-lactate dehydrogenase A chain (LDHA), Ataxia telangiectasia mutated (ATM), 5'-AMP-activated protein kinase catalytic subunit alpha-1 (AMPK), Cellular tumour antigen p53 (p53), Fatty acid synthase (FASN), Long-chain-fatty-acid-CoA ligase 1 (ACSL1), Pyruvate dehydrogenase E1 component subunit alpha (PDHA1).
